# Efficacy and Safety Evaluation of a Novel Low‐Fluence, High‐Frequency Approach for Unwanted Hair Removal

**DOI:** 10.1111/jocd.71138

**Published:** 2026-09-02

**Authors:** Alejandra Fernández Santos, Leticia Huergo Zapico, Susana Valero Freitag

**Affiliations:** ^1^ Termosalud S.L. Gijón, Asturias Spain

**Keywords:** DHR, diode laser, epilation, FDHR, hair removal, wavelength

## Abstract

**Background:**

High‐fluence laser hair removal procedures have provided permanent solutions for unwanted hair growth. However, their association with uncomfortable sessions and adverse effects has prompted the evaluation of new hair removal techniques.

**Objective:**

This study compares the efficacy and safety of a new laser method (linear FDHR; low fluence, high frequency, and controlled movements) with the traditional technique (DHR; high fluence and low frequency), using the Eneka PRO laser device (Termosalud Inc., Gijón, Spain).

**Materials and Methods:**

Thirteen body areas (skin phototypes II–IV) were treated 8 times at 2‐month intervals. DHR mode was applied to one side of the treatment area, whereas linear FDHR mode was delivered on the contralateral side. Efficacy was evaluated 2 months after the final session by digital photographs and hair counts. Safety was assessed during the treatment period and at 6‐month follow‐up visit. Participant satisfaction was assessed via a customized questionnaire.

**Results:**

At the follow‐up, hair clearance rates of the linear FDHR and DHR method (83% and 91%, respectively) were significant (*p* < 0.05) and comparable (*p* > 0.05). Common adverse effects were detected through the treatments and no unexpected adverse effects or long‐term adverse effects occurred. Linear FDHR method was associated with less discomfort (31%) when compared to DHR (54%) (*p* < 0.05). 62% of the participants reported no difference in satisfaction levels between the 2 modes.

**Conclusion:**

The linear FDHR technique is a noninvasive, effective, safe, and well‐tolerated alternative to the traditional method for the removal of unwanted hair.

## 1 Introduction and Objective

Long‐pulsed laser systems for permanent hair reduction have traditionally relied on the combined use of high‐fluence and low‐repetition rates [1, 2, 3]. Delivered through single passes over the treatment area, this approach has proven to be effective in achieving permanent hair reduction [4]. However, the use of high fluences is often associated with increased pain and a higher risk of adverse effects [2].

To overcome these limitations, an alternative concept based on low‐fluence and high‐repetition rates was implemented [5]. This method, commonly referred to as Fast Dynamic Hair Removal (FDHR) or Super Hair Removal (SHR), relies on the gradual accumulation of heat within the hair follicle through the delivery of multiple low‐energy pulses [3, 6, 7, 8]. Instead of inducing immediate photodestruction, this approach exploits the thermal relaxation time (TRT) of the skin and the follicular structures to achieve progressive follicular damage [1, 6].

Despite its improved safety profile, the FDHR technique (hereafter denoted as classic FDHR) is typically performed throughout a randomized movement pattern [2, 3, 9]. This results in an uncontrolled and uneven distribution of energy in the treatment region, potentially leading to local energy overdosing, suboptimal thermal accumulation, and/or inefficient use of the device output [5, 10]. In this context, we propose a novel FDHR technique, based on a controlled linear scanning pattern and referred to as linear FDHR. Unlike the classic randomized approach, this new technique ensures a more uniform and reproducible energy distribution, optimizing heat accumulation within the follicle while improving energy efficiency.

In the present study, we compare the efficacy and safety of the traditional high‐fluence, low‐frequency method (DHR) with the novel linear FDHR approach across different body areas and skin phototypes using the Eneka PRO laser device (Termosalud Inc., Gijón, Spain).

## 2 Materials and Methods

### 2.1 Laser Device

Eneka PRO is a high‐power (up to 5000 W), non‐ablative diode laser platform developed by Termosalud S.L. (Gijón, Spain) for the long‐term reduction of unwanted hair through selective photothermolysis. To adapt to each treatment area characteristics the device incorporates handpieces of different wavelengths (755, 810, 1060 nm, and 808/940/1060 nm) and spot sizes (L, XL and 2XL). The system also offers 2 functioning modes, dynamic hair removal (DHR) and fast dynamic hair removal (FDHR). While the DHR mode delivers individual ultrashort pulses of high power in a low‐to‐medium frequency (up to 4 Hz), FDHR applies multiple pulses of low fluence in a high frequency (up to 10 Hz). According to the characteristics of patient's hair color, skin phototype, and hair thickness, the platform allows the adjustment of the DHR and FDHR technical parameters. By a contact cooling system integrated in the handpiece (sapphire tip) the device reduces cutaneous temperature to prevent thermal skin damage and mitigate treatment discomfort.

### 2.2 Subject Selection

The study comprised 13 treatment areas of 6 healthy volunteers, women (1) and men (5), with ages ranging between 24 and 46 years old (mean age 33.33 years old) and skin phototypes from II to IV. Participants were selected according to inclusion and exclusion criteria. Candidates aged between 18 and 75 years old, with skin phototypes I–VI, no treatment contraindications and no previous laser hair removal procedures in the area to be treated were considered. Exclusion criteria comprised people under 18 years old, over 75 years old and/or those exhibiting any contraindications to laser treatment (Data S1). Written informed consent and authorization for the use of images were obtained from all the included subjects after a complete explanation of the study procedure. The consort flow diagram of the treatment areas included in the study is indicated in Data S2.

### 2.3 Study Protocol

Participants were treated with the Eneka PRO diode laser device, using the 808 nm handpiece with a 20 × 9 mm (1.8 cm^2^) spot size. The subjects received 8 sessions in the chest, abdomen, back, armpits and/or legs at 2‐month intervals. Treatment areas were divided into 2 sides. Each side was randomly assigned to receive either the DHR or the linear FDHR method. DHR procedures were applied by single passes of the handpiece over the entire target region with approximately 25% overlap between treated lines (Data S3). To conduct linear FDHR treatments, the side was further subdivided into grids of approximately 20 cm length. Within each delimited grid, the handpiece was moved 5 times over the same line at a constant speed (approximately 3 s per line), from one end to the other. This process was repeated until the full treatment area was covered with about 25% overlap between lines (Data S3).

Fluence, frequency, and pulse duration parameters for both treatment techniques were established following the device configuration at the first session based on the participant's skin phototype and hair type (color and thickness). Settings were reviewed and adapted in every session according to possible changes in the participants' profiles. DHR fluences and frequencies ranged between 9–27 J/cm^2^ and 1 Hz–3 Hz, respectively. For the linear FDHR treatments, fluences varied from 5 to 8 J/cm^2^ and frequencies from 6 to 10 Hz. According to the selected fluences, pulse duration ranged from AUTO^1^ to 100 ms (Table 1).

**TABLE 1:** Eneka PRO DHR and linear FDHR parameters applied in the treatment sessions.

Working mode	Wavelength	Spot size	Fluence (mean)	Frequency	Pulse duration
DHR	808 nm	1.80 cm^2^	9–27 J/cm^2^ (20.49 J/cm^2^)	1–3 Hz	Auto 30 ms 100 ms
Linear FDHR	808 nm	1.80 cm^2^	5–8 J/cm^2^ (7.30 J/cm^2^)	6–10 Hz	Auto

*Note:* AUTO pulse duration: half of the fluence.

Abbreviations: Hz, hertz; J, joule; ms, milliseconds; nm, nanometers.

Immediately before each laser session, hair was shaved and the participants' eyes were covered with appropriate protective goggles. A 2–3 mm layer of cooling gel was applied in the treatment areas to cool the skin and to ensure good optical coupling between the handpiece and the target tissue and treatment safety. Continuous cooling of the skin was also performed by the cold sapphire tip integrated in the handpiece. No anesthesia was applied to the treatment areas prior to laser sessions.

The evaluation and comparison of the efficacy and safety profiles of both modes, DHR and linear FDHR, were conducted using data taken before starting treatment sessions (baseline), 2 months after the final laser treatment (first follow‐up) and at least 6 months after the last session (final follow‐up). Data collection was performed following the methods described below.

### 2.4 Efficacy Assessment: Hair Clearance Rates

The photographic analysis of the DHR and linear FDHR efficacies was complemented with the estimation of the hair clearance rates. This parameter was calculated by counting the number of hairs at the baseline and at the first follow‐up visit images (2 months after the last session). In the photographs of each treatment area, 6 grids of 1cm^2^ were randomly delimited: 3 for the DHR side and 3 for the linear FDHR region. Grids were placed in identical sites for the before and after photographs. According to the investigators' criteria, 1 evaluator counted the number of hairs in each grid. All structures where the insertion zone to the skin was visible were identified and counted (Data S4). By contrast, to avoid possible double counting, hairs where this region was not identified were not considered. Hair clearance rates were defined using the average values of the 3 counting grids for each working mode. The final parameter was expressed as a percentage.

### 2.5 Photographic Assessment

Standardized photographs of the participants were taken at baseline and at the first follow‐up session (2 months after the last session) to macroscopically evaluate the efficacy of DHR and linear FDHR Eneka PRO procedures. Two types of photographs were taken: general images with a digital camera (*Canon EOS 60D*) and detailed photographs with an *iPad*. For chest, abdomen, and armpit areas, photographs were taken from a frontal perspective, while leg photographs were taken from 2 different perspectives, frontal and back. Before and after images were combined using *Photoshop software* (24.1.1 version). To ensure the correct visual evaluation, both photographs were adjusted in terms of illumination, contrast, and exposure using Photoshop software.

### 2.6 Safety Evaluation: Discomfort (Tolerance), Adverse Events, and Satisfaction Level

Six months after the final laser session (final follow‐up), the participants were asked to fill in a questionnaire to evaluate the safety and the satisfaction profile of the treatments. Post‐treatment discomfort was subjectively quantified according to a visual analogue scale (VAS) ranging from 0 (no pain) to 10 (intolerable pain). The occurrence and severity of long‐term adverse events related to the treatment (erythema, perifollicular edema, blistering, itching sensation, hypo/hyperpigmentation, and/or burns) was reported by the participants in a 10‐grade scale (0, any adverse event and 10, severe adverse event) at the final follow‐up. Immediate adverse events were evaluated by the investigator after each laser session. Finally, participants' satisfaction levels were assessed 2 and 6 months after the last treatment using a 0 (very unsatisfied) to 10 (very satisfied) VAS scale.

### 2.7 Statistical Analysis

Data analysis was performed using the *R software* and the *Rcmdr package* (R version 4.3.02023 and Rcmdr version 2.8‐0). Means, medians, and standard deviations were calculated to describe the improvement after the procedures and to evaluate the discomfort degree, the satisfaction level, and the adverse events occurrence of each epilation mode. Normal distribution assumption was evaluated by *Shapiro–Wilk tests*. Evaluation of discomfort and comparison of hair clearance rates were performed using *t‐*tests for paired samples or Wilcoxon signed‐rank tests for matched pairs. Statistical significance was set at *p* < 0.05.

## 3 Results

### 3.1 Participant Baseline Characteristics and General Results

Thirteen body areas were treated using the Eneka PRO laser device (Table 2). Treatment regions comprised chest (23.08%), abdomen (23.08%), back (15.38%), legs (30.77%), and armpits (7.69%). All participants, 5 men and 1 woman, aged 24 to 46 years old (mean age 33.33) and exhibiting skin phototypes II–IV, successfully completed the 8 laser sessions and the 2‐month and 6‐month follow‐ups. At the 2‐month follow‐up, all the participants exhibited significant hair clearance rates with both techniques.

**TABLE 2:** Demographic characteristics and clinical information of the subjects and the treatment areas included in the clinical trial.

Characteristic | Parameter	Value (range)
Subjects (*n*)	
Men	5
Women	1
Age range (years old)	24–46
Skin phototypes (%)	
II	61.54
III	30.77
IV	7.69
Treatment areas (*n*)	13
Anatomical treatment areas (%)	
Chest	23.08
Abdomen	23.08
Back	15.38
Legs	30.77
Armpits	7.69
Treatment efficacy: hair clearance rates (%)	
DHR	91.30 (48,57–95.59)*
Linear FDHR	83.33 (50–100)*
Treatment discomfort (0–10 VAS scale)	
DHR	3.69*
Linear FDHR	0.77*

* *p* < 0.05.

### 3.2 Hair Count Evaluation

**FIGURE 1:**
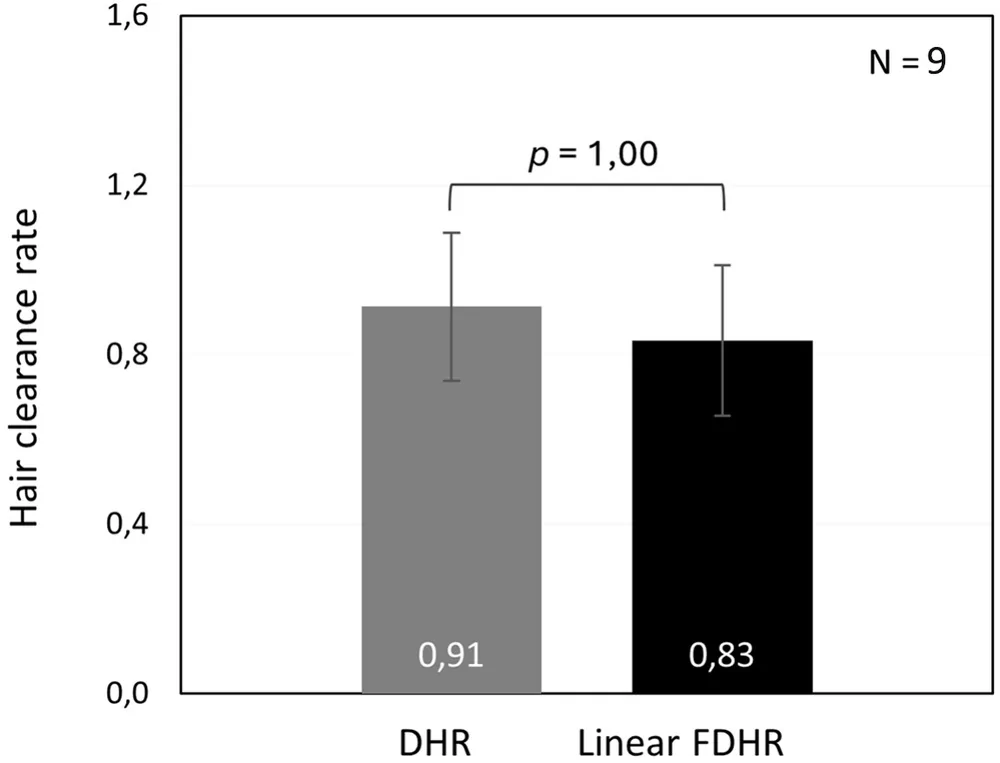
Normalized hair clearance rates for DHR (gray bar) and linear FDHR (black bar) techniques following 8 sessions of Eneka PRO. At the 2‐month follow‐up, DHR achieved 91.30% ± 17.46% (48.57%–95.59%) hair reduction, whereas linear FDHR reached 83.33% ± 17.77% (50%–100%). No significant differences were observed between the techniques (*p* = 1.00; *W* = 22).

Hair clearance rates were calculated in 9 of the 13 treatment areas. The remaining 4 treatment areas were not included in this analysis due to limited image resolution, thereby ensuring an accurate evaluation. At the 2‐month follow‐up visit, significant hair clearance rates of 91.30% ± 17.46% (*p* = 0.0039; *W* = 45) for the DHR technique and 83.33% ± 17.77% (*p* = 8.82 × 10^−7^; *t* = 13.48) for the linear FDHR technique were observed. For DHR, the hair reduction rates ranged from 48.57% to 95.59%. Similarly, linear FDHR hair clearances values varied from 50% to 100%. No statistically significant differences were found between both techniques (*p* = 1.00; *W* = 22) (Figure 1).

### 3.3 Photographic Assessment

The visual analysis of baseline and 2‐month follow‐up photographs showed an overall reduction in hair density across the 13 treated areas with both treatment modalities (Table 3; Data S5). No macroscopic differences were observed between DHR and linear FDHR‐treated sites.

**TABLE 3:** Outcomes in representative treatment regions following Eneka PRO procedures.

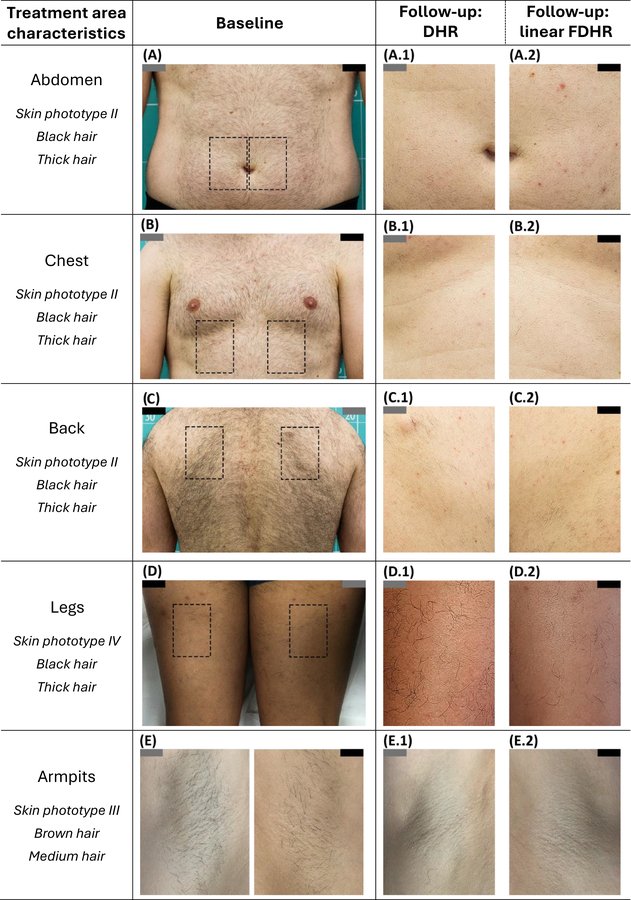

*Note:* The left‐hand column lists the biological characteristics (treatment region, skin phototype, and hair type—color and thickness) of the treated areas. (A–E) Photographs acquired before the start of the epilation treatments are presented in the Baseline column. Hair removal results at the 2‐month follow‐up for (A.1–E.1; gray boxes 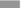) the DHR mode and (A.2–E.2; black boxes 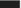) the Linear FDHR technique are shown in the Follow‐up: DHR and Follow‐up: Linear FDHR columns.

### 3.4 Treatment Safety: Discomfort (Tolerance), Adverse Events, and Satisfaction Level

#### 3.4.1 Discomfort (Tolerance)

Tolerance to the linear FDHR and DHR treatments was assessed using a 10‐point VAS scale. The mean discomfort score for the DHR mode was 3.69, corresponding to mild discomfort. Subjects reported no pain or discomfort (Score 0) in 46.15% (*n* = 6) of the treatment areas. Moderate discomfort (Scores 4–6) was reported in 23.08% (*n* = 3) of the DHR areas, whereas 30.77% (*n* = 4) of the treatment regions were associated with severe discomfort (Scores 7–10). The mean discomfort level for the linear FDHR mode was 0.77, indicating no discomfort. Compared to DHR, this value was significantly lower (*p* < 0.05; *W* = 28). In 69.23% (*n* = 9) of the linear FDHR areas no discomfort was reported, while 23.08% (*n* = 3) were associated with mild discomfort (Scores 1–3). Only 7.69% (*n* = 1) reported moderate discomfort. No severe discomfort was reported for linear FDHR treatments.

#### 3.4.2 Adverse Events

Common transient adverse effects were reported immediately after the sessions in 5 out of 13 treatment areas: erythema (mild *n* = 3; moderate *n* = 4; severe = 3), perifollicular edema (moderate *n* = 1; severe = 1), and irritation (mild *n* = 1; moderate *n* = 1; severe *n* = 1). All events were resolved spontaneously without any intervention. No common long‐term adverse events were reported. In addition, there were no unexpected adverse effects, either immediately after the treatments or in the follow‐up sessions (2‐ and 6‐month follow‐ups).

#### 3.4.3 Satisfaction Level

Patient satisfaction was evaluated on a 0–10 VAS scale 2 and 6 months after the last session. At the 2‐month follow‐up, 46.15% (*n* = 6) of the participants were very satisfied with their results, 46.15% (*n* = 6) were satisfied, and 7.69% (*n* = 1) did not see differences. At the 6‐month follow‐up, 7.69% (*n* = 1) and 84.62% (*n* = 11) of the subjects reported being very satisfied and satisfied with the results, respectively. Only 1 of the participants was indifferent to the results (7.69%). Furthermore, when participants were requested to compare the satisfaction levels of both techniques, 61.54% (*n* = 8) did not report any difference; 23.08% (*n* = 3) expressed higher satisfaction level with the DHR technique, and 15.38% (*n* = 2) with the linear FDHR. Finally, regardless of the treatment technique, 100% of the subjects would recommend the procedure to others.

## 4 Discussion

Unwanted hair is a major concern for people seeking aesthetic treatment [4, 11]. As an improvement of temporary epilation methods, diode laser has become the reference gold standard [12]. Traditionally, high‐fluence, low‐frequency laser technique (DHR) has demonstrated high effectiveness across all skin phototypes [3, 4, 13]. However, it has often been associated with pain and adverse effects such as erythema, perifollicular edema, or burns [5, 14, 15, 16]. To overcome these limitations, low‐fluence and high‐frequency protocols with randomized handpiece movements (classic FDHR) were implemented [3, 7, 8], showing comparable efficacy and an improved safety profile [6, 8]. Nevertheless, it has also been associated with operational and handling drawbacks that can potentially compromise treatment efficacy and safety [5]. As it relies on the combination of random movements and prolonged treatment duration, classic FDHR can lead to uneven energy distribution with localized areas of under or overexposure, as well as reduced practitioner comfort. To address the limitations of the 2 techniques, this study introduces a novel linear FDHR method, designed to deliver uniform low‐fluence energy at high frequencies through controlled linear movements.

In our study visual comparison of the baseline and 2‐month follow‐up photographs demonstrated an overall hair density reduction across the 13 treated regions. Macroscopically, no visible differences were detected between the DHR and linear FDHR‐treated contralateral sides. Quantitatively, hair reduction rates were comparable: 91.30% ± 17.46% for the DHR and 83.33% ± 17.77% for the linear FDHR. Although the classic DHR resulted in a slightly higher absolute hair removal percentage, the difference with linear FDHR lacked statistical significance (*p* = 1). Therefore, combined with the absence of visual differences, these values evidenced the therapeutic equivalence of both techniques. These findings are consistent with previous studies comparing high‐fluence and low‐fluence techniques. According to the report of Braun, following 5 sessions, no significant differences in hair reduction were detected (*p* = 0.16). The author reported a 91% hair clearance value for the high‐fluence and 86% for the low‐fluence [13]. Similarly, Koo and colleagues found no significant differences in efficacy between the 2 modes after 5 sessions, reporting hair reduction rates of 52.7% and 57.6% for the high‐fluence and low‐fluence techniques, respectively (*p* = 0.29) [9]. Those differences may be primarily attributed to the use of devices from different generations, whose technical specifications differ in terms of maximum optical power or pulse duration. Furthermore, differences in follow‐up duration may have also contributed to the observed discrepancies, as short‐term follow‐up periods can underestimate the long‐term extent of hair reduction [17].

The equivalence of DHR and linear FDHR modes is based on the distinct biological and thermodynamic mechanisms triggered after their application [5, 6]. The DHR technique relies on the delivery of isolated, high‐fluence pulses that immediately exceed the thermal destruction threshold of hair follicles (≥ 60°C), leading to follicular degeneration [10, 18, 19, 20]. The remaining heat rapidly diffuses into adjacent dermal and epidermal layers, stimulating nociceptors and increasing the risk of severe pain and collateral tissue damage [2, 19, 20]. Conversely, the linear FDHR exploits progressive selective photothermolysis by accumulating subthreshold thermal energy through frequent, low‐fluence pulses [1, 18]. Over time, thelocal thermal threshold is reached, causing irreversible thermal damage to the follicular structures [2, 6, 8, 20]. Considering the Eneka PRO parameters, the linear FDHR mode achieves efficacy levels equivalent to those of the DHR while applying a lower amount of energy to the patient's skin. Whereas conventional DHR delivers approximately 2000 J over a standard treatment area of 100 cm^2^, the linear FDHR mode provides 43.3% less energy (1150 J). Furthermore, the linear FDHR prevents excessive point‐source energy concentration by performing controlled movements [1, 14]. Thus, it restricts extreme thermal spikes into the target tissues, maintaining a stable dermal and epidermal temperature and, consequently, an optimal tolerance profile [6]. In this investigation, the assessment of tolerance levels demonstrated that it was higher in linear FDHR procedures, with treatment described as completely comfortable in 69.23% of the treated areas (VAS score = 0), compared with only 46.15% for the DHR mode (*p* < 0.05; *W* = 28). This tolerance pattern has been reported in previous studies where high‐ and low‐fluence modalities were compared. Using a 10‐grade VAS scale, Koo and colleagues reported significant lower pain scores for the low‐fluence technique (2, 7), compared to the 3, 6 for the high‐fluence one (*p* = 0.0007) [9]. By its part, Braun also determined lower pain ratings in subjects treated with the low‐fluence technique (3, for low‐fluence and 5, for the high‐fluence) (*p* < 0.0001) [13].

Beyond its advantages over DHR, the linear FDHR provides energetic and operational improvements over the classic randomized FDHR protocols. Based on the physical parameters of the Eneka PRO and the different movement patterns, covering a standard 100 cm^2^ area with the linear FDHR technique requires only 9–10 s, 60% less time than the 23.8 s required for classic FDHR. When controlled linear movements are combined with reduced treatment times, the linear FDHR mode can enhance overall treatment maneuverability, reducing the physical discomfort of the practitioner often associated with classic FDHR. In addition, the controlled and repetitive movements of linear FDHR can optimize pulse overlapping and prevent excessive energy accumulation to hair follicles. Thus, linear FDHR ensures a uniform distribution of energy across all hair follicles, maintaining treatment efficacy while minimizing adverse effects occurrence [5]. Uniform movements also guarantee consistent intervals between energy pulse delivery, preventing potential follicular under‐stimulation [10, 21] and ensuring treatment efficacy. In clinical practice, all the previous factors position linear FDHR as a potentially preferred alternative to the classic FDHR and the DHR modes, as it contributes to maximizing thermodynamic efficiency of the treatments, improving treatment safety, and significantly improving practitioner physical comfort.

In conclusion, our outcomes provide clinical evidence demonstrating that delivering low doses of energy at high frequency through a controlled and repetitive linear pattern offers a faster, more comfortable, and more energy‐efficient alternative for laser hair removal procedures. Further comparative trials will be considered to evaluate the method in a larger number of participants and diverse skin phototypes. Nevertheless, the results indicate that the novel linear FDHR technique is a reliable and well‐tolerated alternative for the removal of unwanted hair.

## 5 Conclusions

The findings of this investigation show that the linear FDHR technique, based on the delivery of low fluences at high repetition rates through controlled movements, is a safe, effective, and well‐tolerated procedure that overcomes the drawbacks associated with traditional high‐fluence (DHR) and random low‐fluence (linear FDHR) treatment techniques.

## Author Contributions

All authors, A.F.S., L.H.Z., and S.V.F., contributed to the study performance and to the development of the article: study design, participant recruitment, research monitoring, data collection, data analysis and interpretation, and manuscript drafting. The content of the article was revised and approved by the three authors.

## Ethics Statement

The study, approved with the code CEImPA 2026.010, was conducted in accordance with the criteria established by the Research Ethics Committee of the Principality of Asturias (1964 Declaration of Helsinki and its later amendments).

## Consent

Written informed consent and photographic consent were obtained from all participants for inclusion in the study and publication of their anonymized data.

## Conflicts of Interest

The research team of this study was composed of Termosalud Inc. (Gijón, Spain) employees. The research was supervised by the Asturias Ethics Committee to ensure the right performance of the study and the correct validation of the results obtained. The authors of the paper are responsible for the content of the article.

## Supporting information


**Supporting information:** jocd71138‐sup‐0001‐Supinfo.docx.

## Data Availability

The data that support the findings of this study are available from the corresponding author upon reasonable request.

## Endnotes

^1^
AUTO pulse duration: half of the fluence.
