# Supplementary material for: Efficacy and Safety Evaluation of a Novel Low‐Fluence, High‐Frequency Approach for Unwanted Hair Removal
Source: J Cosmet Dermatol. 2026 Sep 2;25(9):e71138. doi: 10.1111/jocd.71138 (PMC13535483; doi:10.1111/jocd.71138)
Supplement: Supplementary file 1 [file JOCD-25-e71138-s001.docx]

## **Data S1. Eneka PRO^TM^ Contraindications**

- Cancer or tumors
- Pregnancy and breastfeeding
- Use of photosensitizing medication
- People who in the last 6 months have undergone an oral treatment of ROACUTAN, TIGASON, NEOTIGASON (oral Isotrerinoine) or any other type of hormonal treatment.
- Diseases that can be stimulated by 755-1060nm light
- Prolonged exposure to the sun or artificial tanning in the 3 or 4 weeks before or after the treatment.
- Active Herpes Zoster or Simplex infection, chickenpox or contagious impetigo in the treatment area.
- History of keloid scars
- Diabetes mellitus
- Fragile and dry skin
- People who in the last 6 months have subjected the area to be depilated to treatment with CO2 laser, ErbioYag or Dermabrasion.
- Hormonal disorders (which are stimulated under strong light).
- Use of anticoagulants.
- Epilepsy
- History of coagulopathies.
- Immunosuppressive disorders where immunosuppression is evident.

**Data S2. Consort flow diagram of the treatment areas**

*Figure 1.* Flowchart of the subjects involved in the clinical trial (CONSORT).

**Data S3. Application of the Eneka PRO^TM^ techniques**

*Figure 2.* Representative diagram of the 2 Eneka PRO^TM^ laser epilation techniques applied in this research. The DHR mode (left) was performed by the single pass (1x) of the handpiece over the entire treatment area, from one end to the other (grey arrows). Linear FDHR mode (right) was conducted by moving the device handpiece 5 times over the same line (5x), between the ends of areas of approximately 20 cm in length (black arrows). A 25% overlap between treated lines was maintained for both techniques.

**Data S4.- Hair clearance evaluation**

*Figure 3.* Hair follicle counting criteria following Eneka PRO^TM^ treatments. (A) 3 counting grids of 1 cm^2^ were randomly delimited on each treatment area side. Black boxes were used to identify the Linear FDHR side and grey boxes for the DHR side. (B) In each grid, structures with a visible skin insertion zone (thickened area) were identified and counted (black arrowheads). Hairs with no visible thickened area were excluded from the count (red arrowheads) to avoid potential double counting.

**Data S5. Eneka PRO^TM^ treatments: hair removal outcomes**

*Table 1*. Results of Eneka PRO^TM^ treatments in the 13 regions included in this study. For each case, the corresponding biological information (treatment region, skin phototype, and hair type - color and thickness-) is included. Letters A-M present the images taken before the treatments (Baseline). The 2 columns on the right show the follow-up results after 8 treatment sessions using the DHR mode (A.1 - M.1; grey boxes	) and the Linear FDHR mode (A.2 - M.2; black boxes	).

| **BASELINE** | **2-MONTH FOLLOW - UP** | |
| --- | --- | --- |
|  | **DHR mode** | **Linear FDHR mode** |
| **Abdomen treatment:** Skin phototype II. Black and thick hair | | |
|  |  | |
| **Chest treatment:** Skin phototype II. Black and thick hair | | |
|  |  | |
| **Back treatment** Skin phototype II. Black and thick hair | | |
|  |  | |

*Table 1*. Continued

| **BASELINE** | **2-MONTH FOLLOW - UP** | |
| --- | --- | --- |
|  | **DHR mode** | **Linear FDHR mode** |
| **Leg treatment:** Skin phototype IV. Black and thick hair | | |
|  |  | |
| **Armpit treatment:** Skin phototype III. Brown and medium thickness hair | | |
|  |  | |
| **Leg treatment:** Skin phototype II. Brown and fine hair | | |
|  |  | |
| **Chest treatment:** Skin phototype III. Black and medium thickness hair | | |
|  |  | |

*Table 1*. Continued

| **BASELINE** | **2-MONTH FOLLOW - UP** | |
| --- | --- | --- |
|  | **DHR mode** | **Linear FDHR mode** |
| **Leg treatment:** Skin phototype III. Brown and medium thickness hair | | |
|  |  | |
| **Abdomen treatment:** Skin phototype II. Black and thick hair | | |
|  |  | |
| **Leg treatment:** Skin phototype II. Black and thick hair | | |
|  |  | |
| **Back treatment:** Skin phototype II. Black and thick hair | | |
|  |  | |

*Table 1*. Continued

| **BASELINE** | **2-MONTH FOLLOW - UP** | |
| --- | --- | --- |
|  | **DHR mode** | **Linear FDHR mode** |
| **Chest treatment:** Skin phototype II. Black and thick hair | | |
|  |  | |
| **Abdomen treatment**: Skin phototype III. Black and fine thickness hair | | |
|  |  | |
